# Association Between Neonatal Thyroid Function and Anogenital Distance from Birth to 48 Months of Age

**DOI:** 10.3389/fendo.2021.736505

**Published:** 2021-09-08

**Authors:** Min Luan, Hong Liang, Guanghong Fang, Ziliang Wang, Xiujuan Su, Aimin Chen, Maohua Miao, Wei Yuan

**Affiliations:** ^1^National Health Commission (NHC) Key Lab. of Reproduction Regulation (Shanghai Institute for Biomedical and Pharmaceutical Technologies), School of Public Health, Fudan University, Shanghai, China; ^2^NHC Key Lab. of Reproduction Regulation (Shanghai Institute for Biomedical and Pharmaceutical Technologies), Fudan University, Shanghai, China; ^3^Clinical Research Center, Shanghai First Maternity and Infant Hospital, Tongji University School of Medicine, Shanghai, China; ^4^Department of Biostatistics, Epidemiology and Informatics, Perelman School of Medicine, University of Pennsylvania, Philadelphia, PA, United States

**Keywords:** neonatal thyroid function, thyroid hormones, anogenital distance, anogenital index, cohort study

## Abstract

**Background:**

Evidence from animal studies has indicated that neonatal thyroid function is vital for the reproductive development. Anogenital distance (AGD), a sensitive biomarker of the fetal hormonal milieu, can be used to predict adult reproductive disorders. However, few human studies have examined the association between neonatal thyroid function and AGD. We aimed to explore their associations in a birth cohort study.

**Methods:**

Concentrations of thyroid stimulating hormone (TSH) and thyroid hormones (THs), including total triiodothyronine (TT_3_), total thyroxine (TT_4_), free triiodothyronine (FT_3_), and free thyroxine (FT_4_) were measured in cord plasma in the Shanghai-Minhang Birth Cohort. The offspring AGD (AGD_AP_ [anus–penis] and AGD_AS_ [anus–scrotum] for boys and AGD_AC_ [anus–clitoris] and AGD_AF_ [anus–fourchette] for girls), body weight and anogenital index (AGI = AGD/weight [mm/kg]) were obtained at each follow-up visit. In total, 344 children (194 boys and 150 girls) with cord plasma concentrations of THs and TSH and at least one AGD measurement at birth and at 6, 12, and 48 months of age were included. Multiple linear regression and generalized estimating equation (GEE) models were used to examine the associations of cord plasma concentrations of THs and TSH with AGI.

**Results:**

Multiple linear regression models showed inverse associations of TT_4_, FT_3_, and FT_4_ with female AGI, although statistical significance was only reached at birth, 6 and 48 months of age. These associations were also found in GEE models: higher TT_4_ and FT_4_ concentrations were associated with lower AGI_AC_ (TT_4_: β = -0.27, 95% CI: -0.50, -0.03 for middle *vs*. lowest tertile; FT_4_: β = -0.38, 95% CI: -0.61, -0.16 for middle and β = -0.30, 95% CI: -0.55, -0.04 for highest *vs*. lowest tertile). Besides, girls with the highest tertile of FT_3_ concentrations had lower AGI_AF_ than those with the lowest tertile (the highest *vs*. lowest tertile: β = -0.22, 95% CI: -0.36, -0.08). Positive associations between TSH and AGI at birth and at 12 months of age were observed in boys.

**Conclusions:**

This study provides further evidence on the effects of neonatal thyroid function on reproductive development at an early life stage.

## 1 Introduction

Accumulating evidence from animal studies has indicated that neonatal thyroid function influences gonadal differentiation and reproductive function (1–4). Administration of large doses of thyroxine to neonatal female rats has been shown to delay vaginal opening and first estrus (5). Neonatal 3,5,3’-triiodothyronine excess in male rats was associated with a decrease in adult testis size (6), and might promote the apoptosis of germ cells in the neonatal testis (7). In humans, thyroid dysfunction is associated with decreased semen quality, decreased sexual activity, menstrual disturbances, and infertility (8–11). However, limited studies have evaluated the influence of thyroid function starting from birth on reproductive development since the information on reproductive development in early life is difficult to obtain, and decades may lapse between neonatal thyroid function and some manifest reproductive end points such as puberty development.

Anogenital distance (AGD), the distance from the center of the anus to the genital tubercle, has been widely recognized as a sensitive biomarker of the fetal hormonal milieu, and a measure of reproductive toxicity in animal models (12). Measurements of AGD at birth and during early childhood are also known to track through adulthood and can be used to predict various reproductive health disorders in adult humans (13, 14). Shorter AGD has been found to be associated with poor sperm quality (15), infertility (16), and lower testosterone concentrations (16) in men and associated with increased risk of endometriomas and deep infiltrating endometriosis in women (17). To date, only one human study has assessed the associations of thyroid stimulating hormone (TSH), free triiodothyronine (FT_3_), and free thyroxine (FT_4_) concentrations in umbilical cord serum with AGD at birth (18), indicating that thyroid function may be involved in male gonadal development in early life stages. However, its longitudinal effects at a later age are unknown.

In the present study, we examined the longitudinal associations between neonatal thyroid function, as reflected by the concentrations of TSH and thyroid hormones (THs, including total triiodothyronine (TT_3_), total thyroxine (TT_4_), FT_3_, and FT_4_)) in cord plasma, and repeated AGD measurements from birth to 48 months of age.

## 2 Materials and Methods

### 2.1 Study Design and Population

The Shanghai-Minhang Birth Cohort Study (S-MBCS) is an ongoing prospective cohort study designed to determine the distributions of a wide spectrum of maternal environmental exposures and examine their effects on pregnant women and their children (19, 20). From April to December 2012, pregnant women were recruited during their first prenatal care visit (at 12–16 weeks of gestation) at Maternal and Child Health Hospital of Minhang district in Shanghai, China. Briefly, women were eligible for inclusion if they were registered residents of Shanghai, had no history of hospital-diagnosed chronic disease, planned to be deliver their baby in the study hospital, and were willing to attend specified interviews during pregnancy and after delivery. In total, 1,292 eligible pregnant women agreed to participate in this study and completed a structured questionnaire at enrollment.

**Figure 1:**
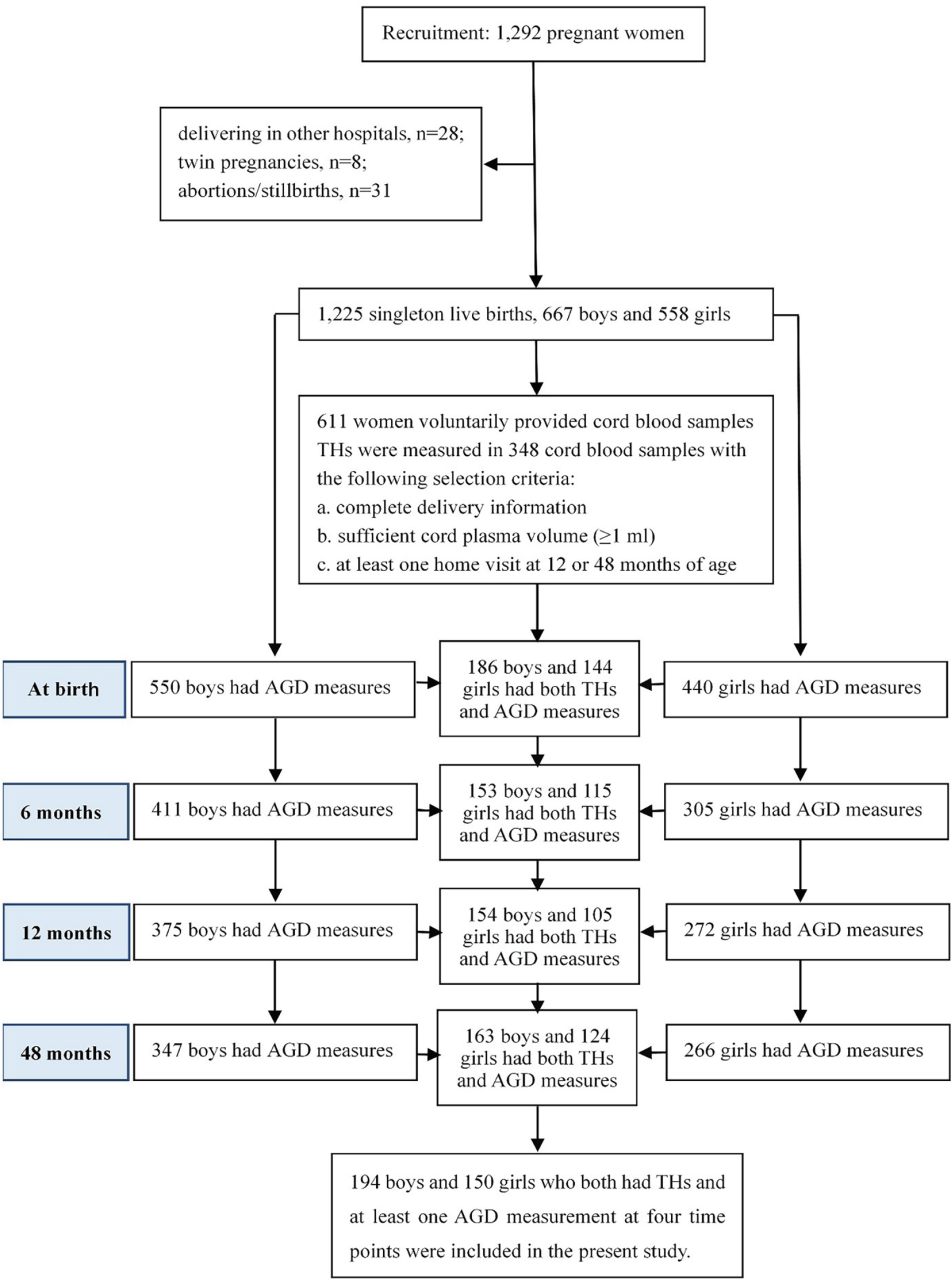
Study population of the present study from the Shanghai-Minhang Birth Cohort Study.

At delivery, 1,225 participants gave singleton live births, including 667 (54.4%) boys and 558 (45.6%) girls. A total of 611 women voluntarily provided cord blood samples during delivery. Due to limited funding, concentrations of THs and TSH were measured in a subset of 348 cord blood samples of infants with complete information at delivery, sufficient cord plasma volume (≥1 ml), and at least one follow-up at 12 or 48 months of age. Among them, two AGD metrics were measured for 186, 153, 154, and 163 boys, and for 144, 115, 105, and 124 girls at birth, 6, 12 and 48 months of age, respectively. Ultimately, 344 children (194 boys and 150 girls) who both have cord plasma concentrations of THs and TSH and at least one AGD measurement at the four time points were included in the present study, as shown in **Figure 1**.

The study protocol was approved by the ethical committee of Shanghai Institute of Planned Parenthood Research. All women provided informed consent for themselves and their children before participating in the S-MBCS and postnatal follow-up visits.

### 2.2 Measurement of Neonatal Thyroid Function

Cord blood samples were collected from the umbilical vein at the time of birth. The samples were centrifuged to retrieve plasma, which was immediately frozen at −80°C until shipment to the clinical laboratory of the affiliated hospital of Shanghai Institute of Planned Parenthood Research for the measurement of THs and TSH concentrations. An Electrochemiluminescence immunoassay with kits obtained from Roche Cobas e601 analyzer was performed to measure the concentrations of TSH, TT_4_, FT_4_, TT_3_, and FT_3_ (21). The analytical sensitivity was 0.3 nmol/L, 5.4 nmol/L, 0.4 pmol/L, 0.3 pmol/L, and 0.27 μIU/ml for TT_3_, TT_4_, FT_3_, FT_4_, and TSH, respectively. We also measured thyroid peroxidase antibody (TPOAb) concentration. TPOAb positivity was defined as a TPOAb concentration of ≥ 34 IU/L.

### 2.3 Measurement of AGD

The methods and procedures for the measurement of AGD have been described in detail elsewhere (22–24). In girls, AGD_AC_ was measured from the center of the anus to the anterior surface of the clitoral hood, and AGD_AF_ was measured from the center of the anus to the posterior end of the fourchette. In boys, AGD_AP_ was measured from the center of the anus to the anterior base of the penis where the penile tissue met the pubic bone, and AGD_AS_ was measured from the center of the anus to the posterior base of the scrotum where the skin changed from rugate to smooth. All children were placed in a dorsal decubitus position with both legs held back in a frog leg position to obtain accurate AGD measurements. Before each follow-up visit, we conducted standardized training to ensure the accuracy of AGD measurements. AGD measurements were conducted using the same Vernier caliper with increments of 0.1 mm in children during their first 3 days of life and at 6 months ± 2 weeks, 12 months ± 2 weeks, and 48 months ± 2 weeks of age by the same four trained examiners. To evaluate the inter-examiner variability, two examiners took independent measurements on each newborn on the same day using the same method in 15 girls and 15 boys. The intra-class correlation coefficients of AGD_AP_, AGD_AS_, AGD_AC_ and AGD_AF_ were 0.836, 0.731, 0.624, and 0.722, respectively, indicating moderate-to-good inter-rater reliability for the AGD measurements (25). The examiners who participated in the AGD measurements had no knowledge regarding the neonatal THs or TSH concentrations.

### 2.4 Covariates

The demographic characteristics, health conditions, medical and reproductive histories, and lifestyle factors (e.g., smoking and drinking habits) of pregnant women and their partners were obtained at enrollment through a structured questionnaire. After delivery, information on infant sex, birth weight, date of birth, mode of delivery, and gestational age was extracted from the electronic medical records of the study hospitals. Feeding practices and additional information were obtained from questionnaires filled by the mother or another caregiver at each postnatal follow-up visit.

### 2.5 Statistical Analyses

The distributions of participant characteristics were summarized as mean (standard deviation, SD) for continuous variables or counts and percentages for categorical variables. The distributions of TT_3_, TT_4_, FT_3_, and FT_4_ concentrations were normal, whereas that of TSH concentrations were skewed. Thus, TSH concentrations were log_10_-transformed for further analyses. Since AGD was reported to be dependent on body weight, we used the anogenital index [AGI = AGD/weight (mm/kg)] as a weight-normalized index of AGD, as reported previously (26). We also used mean (SD), and percentiles to describe the distributions of AGI at birth and at 6, 12, and 48 months of age and to describe the distributions of neonatal TT_3_, TT_4_, FT_3_, FT_4_, and TSH concentrations.

#### 2.5.1 Main Analyses of the Association Between Neonatal THs and TSH and AGI

A generalized additive model (GAM) was used to examine the nonlinearity assumptions between neonatal THs and log_10_-transformed TSH concentrations and AGI. As non-linear associations were observed from GAM, the concentrations of TT_3_, TT_4_, FT_3_, FT_4_, and log_10_-transformed TSH were treated as categorical variables (by tertiles) in the analyses. We first performed multiple linear regression models at each visit to assess the associations of THs and TSH concentrations with AGI. To take advantage of the longitudinal design and repeated measurements of AGD, generalized estimating equation (GEE) models were then applied to estimate β coefficients and 95% confidence intervals (CIs) for neonatal THs and TSH concentrations in relation to AGI after accounting for correlations of repeated AGD measures that had been obtained from four visits. We also added child age at follow-up into the GEE model and entered the interaction terms between THs and TSH concentrations (categorical) and child age (categorically) to assess differences in the associations over time. Since most interaction terms were not statistically significant (*P* > 0.1, **Table S1**), overall estimates were provided for AGI at 0–4 years of age.

Potential confounders were selected based on prior knowledge (27) or the change-in-estimate principle. For the latter criterion, the covariates were also retained in the final analyses if they caused a ≥10% change in the effect estimates for the associations of THs and TSH concentrations with AGI. Maternal education was also considered as a covariate since it is a good measure of socio-economic status (28) which can also indicate some often unobservable characteristics (29), and women’s socio-economic status were associated with the health endpoints of children (29, 30). Potential confounders finally considered in the models were maternal age at conception (years), maternal education (high school or below/college or above), maternal pre-pregnancy body mass index (BMI: <18.5, 18.5–24, and ≥24 kg/m^2^), maternal pre-pregnancy passive smoking (yes/no), paternal alcohol consumption before conception (yes/no), and gestational weeks (weeks).

#### 2.5.2 Sensitivity Analyses

First, in addition to AGI, we used AGD as the dependent variable, and adjusted for weight-for-length z-scores in the GEE models to facilitate comparison with other studies (18). Second, TPOAb positivity is associated with a higher risk of developing autoimmune thyroiditis in children and adolescents (31), which may have adverse effects on reproductive outcomes (32). We thus excluded the subjects with TPOAb positivity (four girls and three boys) to test the robustness of the results. Third, to reduce the potential influence of incomplete neonatal thyroid development, we excluded subjects with gestational age <37 weeks. In addition, considering the significant variation in neonatal TSH and THs concentrations across different modes of delivery and maternal BMI (33–35), we re-ran the analyses in children who were delivered vaginally and whose mothers were of normal weight (18.5 < BMI < 24 kg/m^2^). We also re-ran the final analyses after excluding six children whose mothers reported prior or current diagnoses of any thyroid-related disease (e.g., autoimmune thyroid diseases, thyroid nodules, thyroid cancer, and thyroiditis)/any related medication use at enrollment to evaluate whether maternal thyroid-related disease/related medication use could affect the reliability of our results.

SAS 9.4 (SAS Institute Inc., Cary, NC, USA) was used for statistical analyses. A two-tailed value of *p* < 0.05 was considered statistically significant.

## 3 Results

### 3.1 Participant Characteristics

The characteristics of the 344 mother–child pairs are presented in **Table 1**. The mean (SD) age of mothers at conception was 28.09 (3.38) years and the mean (SD) gestational age was 39.67 (1.18) weeks. Most of mothers were Han Chinese (97.66%), nulliparous (86.88%), received a college degree or higher (78.13%), reported a monthly per capita household income >4000 RMB (79.53%), and had normal weight before pregnancy (72.78%). In addition, 58.31% mothers reported that they were not exposed to passive smoking before pregnancy, and 67.44% fathers reported no alcohol consumption within 3 months before conception. All newborns had a normal 5-min Apgar score (≥8). There was no statistically significant difference in demographic characteristics between the included mother–child pairs and those excluded (**Table S2**).

**Table 1:** Descriptive characteristics of the study participants.

Characteristics	All subjects	Girls (n=150)	Boys (n=194)
	Mean (SD)/N (%)	Mean (SD)/N (%)	Mean (SD)/N (%)
Maternal age (years)	28.09 (3.38)	28.08 (2.97)	28.11 (3.67)
Gestational age (weeks)	39.67(1.18)	39.70 (1.15)	39.64 (1.2)
Pre-pregnancy body mass index (kg/m²)			
<18.5	68 (20.12)	29 (19.73)	39 (20.42)
18.5-24	246 (72.78)	112 (76.19)	134 (70.16)
≥24	24 (7.10)	6 (4.08)	18 (9.42)
Educational level			
High school or below	75 (21.87)	29 (19.33)	46 (23.83)
College or above	268 (78.13)	121 (80.67)	147 (76.17)
Parity^*^			
Nulliparous	298 (86.88)	137 (91.33)	161 (83.42)
Multiparous	45 (13.12)	13 (8.67)	32 (16.58)
Race			
Han	334 (97.66)	145 (96.67)	189 (98.44)
Others	8 (2.34)	5 (3.33)	3 (1.56)
Family income per capita (RMB)			
<4000	70 (20.47)	29 (19.33)	41 (21.35)
4000-8000	151 (44.15)	70 (46.67)	81 (42.19)
≥8000	121 (35.38)	51 (34)	70 (36.46)
Maternal pre-pregnancy passive smoking			
Yes	143 (41.69)	66 (44.3)	77 (39.69)
No	200 (58.31)	83 (55.7)	117 (60.31)
Paternal alcohol consumption before conception			
Yes	112 (32.56)	46 (30.67)	66 (34.02)
No	232 (67.44)	104 (69.33)	128 (65.98)
5-min Apgar score			
8	1 (0.29)	1 (0.67)	0
9	341 (99.13)	149 (99.33)	192 (98.97)
10	2 (0.53)	0	2 (1.03)
Weight at corresponding ages			
Birth (g)	3449.38 (427.95)	3354 (423.6)	3523 (417.62)
6 months (kg)	8.44 (1.03)	7.96 (0.94)	8.80 (0.94)
12 months (kg)	10.39 (1.12)	9.90 (0.99)	10.77 (1.08)
48 months (kg)	17.94 (2.44)	17.41 (2.34)	18.34 (2.44)

Missing data: maternal education (n = 1), maternal pre-pregnancy BMI (n = 6), maternal passive smoking (n = 1), parity (n = 1), race (n=2), and family income per capita (n = 2).

^*^Significance level of the chi-square test.

### 3.2 Distributions of AGI

All AGI measures were normally distributed. In girls, the mean (SD) AGI_AC_ was 8.75 (1.26), 4.52 (0.92), 4.06 (0.73), and 3.25 (0.67) mm/kg and the mean (SD) AGI_AF_ were 2.53 (0.70), 1.80 (0.58), 1.71 (0.52), and 1.78 (0.42) mm/kg at birth, 6 months of age, 12 months of age, and 48 months of age, respectively. In boys, the mean (SD) AGI_AP_ was 11.73 (1.54), 7.50 (1.25), 6.49 (1.09), and 5.29 (0.68) mm/kg and the mean (SD) AGI_AS_ was 4.32 (1.13), 3.10 (1.19), 2.79 (0.87), and 2.49 (0.53) mm/kg at birth, 6 months of age, 12 months of age, and 48 months of age, respectively (**Table 2**). The distributions of AGD from birth to 48 months of age are also shown in **Figure S1**.

**Table 2:** Distributions of AGI and neonatal THs and TSH concentrations.

	Mean (SD)	Percentiles				
		Min	25th	50th	75th	Max
AGI_AC_ (mm/kg)						
Birth	8.75 (1.26)	5.85	7.90	8.63	9.44	13.37
6 months	4.52 (0.92)	2.35	3.94	4.52	5.10	7.54
12 months	4.06 (0.73)	2.45	3.50	3.95	4.41	6.26
48 months	3.25 (0.67)	1.96	2.76	3.13	3.64	5.64
AGI_AF_ (mm/kg)						
Birth	2.53 (0.70)	0.71	2.09	2.63	2.99	4.32
6 months	1.80 (0.58)	0.72	1.38	1.65	2.03	3.79
12 months	1.71 (0.52)	0.65	1.28	1.60	2.03	3.26
48 months	1.78 (0.42)	1.03	1.50	1.74	1.96	3.44
AGI_AP_ (mm/kg)						
Birth	11.73 (1.54)	8.58	10.42	11.72	12.72	16.23
6 months	7.50 (1.25)	4.78	6.61	7.38	8.17	11.67
12 months	6.49 (1.09)	4.10	5.71	6.35	7.19	9.68
48 months	5.29 (0.68)	3.78	4.78	5.38	5.77	6.75
AGI_AS_ (mm/kg)						
Birth	4.32 (1.13)	1.89	3.49	4.22	4.92	7.91
6 months	3.10 (1.19)	1.02	2.23	3.05	3.92	6.25
12 months	2.79 (0.87)	0.92	2.28	2.74	3.26	5.28
48 months	2.49 (0.53)	1.52	2.08	2.44	2.84	4.22
TT_3_ (nmol/L)	0.86 (0.16)	0.10	0.77	0.85	0.94	1.63
TT_4_ (nmol/L)	94.12 (26.63)	33.54	77.23	93.07	109.60	183.20
FT_3_ (pmol/L)	1.82 (0.37)	1.11	1.55	1.78	2.01	3.55
FT_4_ (pmol/L)	14.22 (1.94)	9.62	12.90	14.17	15.39	20.92
TSH (uIU/mL)	6.47 (0.21) *	1.28	4.32	6.46	9.48	38.52

*GM (GSD) Log_10_-transformed.

### 3.3 Distributions and Determinants of THs and TSH

In all subjects, the mean concentrations of TT_3_, TT_4_, FT_3_, FT_4_ and TSH in cord plasma were 0.86 nmol/L, and 94.12 nmol/L, 1.82 pmol/L, 14.22 pmol/L, and 6.47 uIU/mL, respectively, as shown in **Table 2**.

In univariate analyses (**Table S3**), children whose parents were younger than 25 years, whose fathers drank alcohol within 3 months before conception, and who were delivered vaginally (*vs*. cesarean section) had significantly higher neonatal TSH concentrations. Additionally, children born to mothers who were underweight (*vs*. normal weight) and gave birth *via* vaginal delivery had lower FT_3_ and FT_4_ concentrations. Further, children born to mothers who were exposed to passive smoking before pregnancy had higher FT_3_ and FT_4_ concentrations and children born to fathers who drank alcohol within 3 months before conception had higher FT_3_ concentrations.

### 3.4 Associations of Neonatal THs and TSH Concentrations With AGI in Girls

**Figure 2:**
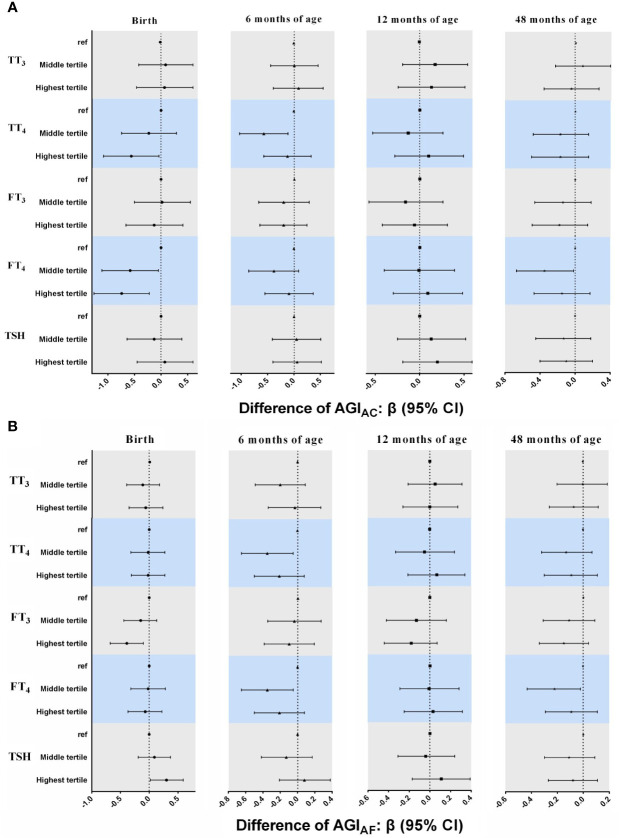
Associations between neonatal TH and TSH concentrations and AGI_AC_ and AGI_AF_ in girls from birth to 48 months of age. In multiple linear regression models. **(A)** AGI_AC_ [anus-clitoris] **(B)** AGI_AF_ [anus-fourchette]. All models adjusted for maternal age, maternal education, maternal pre-pregnancy BMI, gestational weeks, maternal passive smaoking, and paternal alcohol comsumption.

In general, multiple linear regression models showed that AGI_AC_ and AGI_AF_ in girls tended to be lower in the higher tertile groups of TT_4_, FT_4_, and FT_3_ in cord plasma than those of girls in the lowest tertile, although statistical significance was not reached for all time points (**Figure 2**). Girls who had higher concentrations of TT_4_ had lower AGI_AC_ at birth (β = -0.56, 95% CI: -1.08, -0.04 for highest *vs*. lowest tertiles) and 6 months of age (β = -0.57, 95% CI: -1.03, -0.11 for middle *vs*. lowest tertiles), and lower AGI_AF_ at 6 months of age (β = -0.35, 95% CI: -0.65, -0.06 for middle *vs*. lowest tertiles). Higher FT_4_ concentrations were associated with lower AGI_AC_ at birth (β = -0.58, 95% CI: -1.11, -0.05 for middle and β = -0.74, 95% CI: -1.26, -0.22 for highest *vs*. lowest tertiles) and 48 months of age (β = -0.35, 95% CI: -0.67, -0.02 for middle *vs*. lowest tertiles), and with lower AGI_AF_ at 6 months of age (β = -0.35, 95% CI: -0.65, -0.05 for middle *vs*. lowest tertiles) and 48 months of age (β = -0.22, 95% CI: -0.43, -0.02 for middle *vs*. lowest tertiles). In addition, girls in the highest tertile of FT_3_ concentrations had lower AGI_AF_ at birth (β = -0.39, 95% CI: -0.68, -0.10) than those in the lowest tertile.

Similar inverse associations of TT_4_, FT_4_, and FT_3_ with AGI in girls were also found in the GEE models (**Table 3**). Higher neonatal TT_4_ and FT_4_ concentrations were associated with lower AGI_AC_ (TT_4_: β = -0.27, 95% CI: -0.50, -0.03 for middle *vs*. lowest tertiles; FT_4_: β = -0.38, 95% CI: -0.61, -0.16 for middle and β = -0.30, 95% CI: -0.55, -0.04 for highest *vs*. lowest tertiles). Additionally, girls in the highest tertile of FT_3_ concentrations had lower AGI_AF_ than those in the lowest tertile (β = -0.22, 95% CI: -0.36, -0.08).

**Table 3:** Associations between TH and TSH concentrations in cord plasma and AGI from birth to 48 months of age in GEE models.

THs and TSH	Girl		Boy	
	AGI_AC_	AGI_AF_	AGI_AP_	AGI_AS_
TT_3_				
Lowest tertile	Ref	Ref	Ref	Ref
Middle tertile	0.06 (-0.17, 0.29)	-0.07 (-0.22, 0.07)	0.08 (-0.17, 0.34)	0.16 (-0.07, 0.39)
Highest tertile	0.05 (-0.21, 0.31)	-0.04 (-0.19, 0.11)	0.15 (-0.13, 0.43)	0.13 (-0.10, 0.37)
TT_4_				
Lowest tertile	Ref	Ref	Ref	Ref
Middle tertile	-0.27 (-0.50, -0.03)^*^	**-0.14 (-0.29, 0.02)^#^**	-0.17 (-0.45, 0.10)	-0.07 (-0.32, 0.17)
Highest tertile	**-0.22 (-0.46, 0.01)^#^**	-0.09 (-0.24,0.07)	0.01 (-0.28, 0.29)	0.03 (-0.19, 0.24)
FT_3_				
Lowest tertile	Ref	Ref	Ref	Ref
Middle tertile	-0.10 (-0.35, 0.14)	-0.09 (-0.24, 0.06)	0.05 (-0.22, 0.32)	0.08 (-0.15, 0.32)
Highest tertile	-0.16 (-0.41, 0.09)	-0.22 (-0.36, -0.08)^*^	-0.01 (-0.28, 0.27)	-0.04 (-0.28, 0.19)
FT_4_				
Lowest tertile	Ref	Ref	Ref	Ref
Middle tertile	-0.38 (-0.61, -0.16)^*^	**-0.15 (-0.31, 0.01)^#^**	-0.11 (-0.37, 0.15)	-0.07 (-0.30, 0.16)
Highest tertile	-0.30 (-0.55, -0.04)^*^	-0.11 (-0.27, 0.06)	-0.01 (-0.30, 0.29)	-0.01 (-0.26, 0.24)
TSH (log 10-transformed)				
Lowest tertile	Ref	Ref	Ref	Ref
Middle tertile	-0.04 (-0.27, 0.19)	-0.04 (-0.19, 0.11)	0.08 (-0.22, 0.37)	0.16 (-0.08, 0.40)
Highest tertile	0.05 (-0.22, 0.32)	0.11 (-0.05, 0.27)	0.14 (-0.15, 0.43)	0.23 (0.0005, 0.46)^*^

Adjusted for maternal age, maternal education, maternal pre-pregnancy BMI, gestational weeks, maternal passive smoking, paternal alcohol consumption, and child’s age (categorized as birth, 0.5, 1, 2, and 4 years).

^*^Statistically significant difference (p < 0.05).

^#^Marginally significant difference (p < 0.10).

### 3.5 Associations of Neonatal THs and TSH Concentrations With AGI in Boys

**Figure 3:**
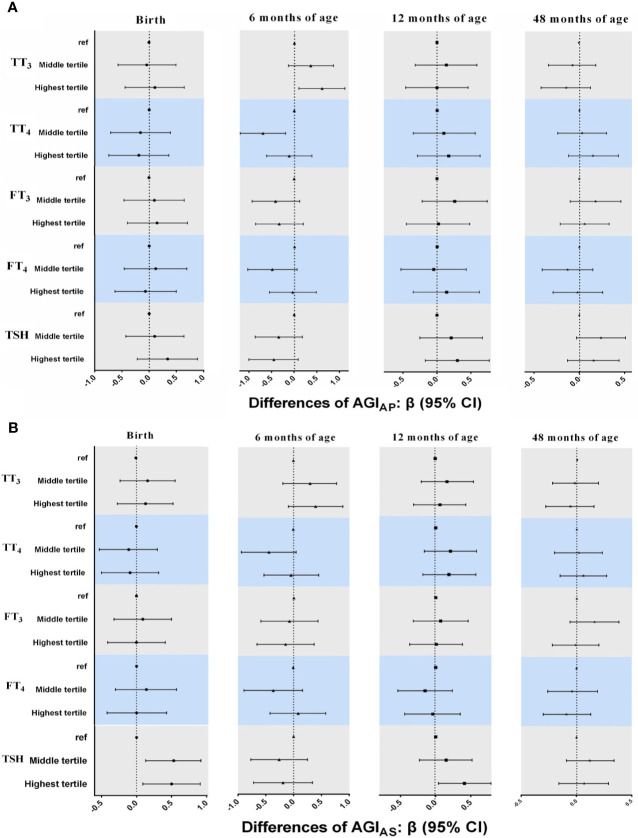
Associations between neonatal TH and TSH concentrations and AGI_AP_ and AGI_AS_ in boys from birth to 48 months of age. in multiple linear regression models. **(A)** AGI_AP_ [anus-penis] **(B)** AGI_AS_ [anus-scrotum]. All models adjusted for maternal age, maternal education, maternal pre-pregnancy BMI, gestational weeks, maternal passive smaoking, and paternal alcohol comsumption.

For THs, the pattern of the results in multiple linear regression models (**Figure 3**) was inconsistent from birth to 48 months of age, although there were a positive association between TT_3_ concentrations and AGI_AP_ at 6 months of age (β = 0.61, 95% CI: 0.10, 1.11 for highest *vs*. lowest tertiles) and an inverse association between TT_4_ concentrations and AGI_AP_ at 6 months of age (β = -0.69, 95% CI: -1.19, -0.19 for middle *vs*. lowest tertiles).

Boys in the higher tertile of log_10_-transformed TSH had higher AGI_AS_ at birth (β = 0.53, 95% CI: 0.13, 0.92 for middle and β = 0.50, 95% CI: 0.09, 0.91 for highest *vs*. lowest tertiles) and 12 months of age (β = 0.41, 95% CI: 0.04, 0.79 for highest *vs*. lowest tertiles; **Figure 3**). The positive association between TSH concentrations and AGI_AS_ remained in GEE models (β = 0.23, 95% CI: 0.0005, 0.46 for highest *vs*. lowest tertiles; **Table 3**), but the interaction term between TSH and child age at follow-up was statistically significant, indicating that the association between TSH and AGI_AS_ may change over time (*P* < 0.1; **Table S2**).

### 3.6 Sensitivity Analyses

We conducted several sensitivity analyses using GEE models. When we used AGD instead of AGI as the dependent variable, inverse associations between concentrations of TT_4_, FT_3_, and FT_4_ and AGD were also observed in girls, but the association between TSH concentrations and AGD in boys became non-significant in GEE models (**Table S4**). The results were virtually identical in sensitivity analyses after excluding infants with TPOAb positivity, with gestational age <37 weeks, or those whose mothers reported prior or current diagnoses of thyroid-related disease/related medication use, and after restricting girls whose mothers had a normal weight (**Tables S5–S8**). Similar patterns were also observed in girls who were delivered vaginally, although some associations were not statistically significant, possibly due to the small sample size (**Table S9**).

## 4 Discussion

The present study is the first longitudinal assessment of the association between neonatal thyroid function and AGD from birth to 48 months of age. Higher neonatal concentrations of TT_4_, FT_3_, and FT_4_ were associated with lower AGI in girls at 0–48 months of age; however, no consistent pattern for THs was found in boys. Positive associations between TSH concentrations and AGI at birth and at 12 months of age were observed in boys.

Numerous epidemiological studies have reported that alterations in neonatal THs and TSH concentrations are associated with infant neurodevelopment (21, 36) and physical growth (18, 37). However, evidence on the effects of THs on reproductive development is limited. Only one human study has assessed the effects of FT_3_, FT_4_, and TSH concentrations in cord serum on AGD at birth (18). In female newborns, the overall patterns of association between higher FT_3_ and FT_4_ and shorter AGD were observed in both our study and the study by Liu et al. (18), although the association was not statistically significant in the study by Liu et al. These differences are likely due to different neonatal TH concentrations (mean comparison: 1.82 *vs*. 1.63 for FT_3_; 14.22 *vs*. 15.61 for FT_4_). The concentrations of THs in the previous study were measured in cord serum and not in cord plasma, and the laboratory method used was also different (18). In line with the study by Liu et al. (18), we found a positive association between TSH concentration and AGI_AS_ in male newborns. We also found a positive association between TSH and AGI_AS_ at 12 months of age. Given that TSH concentrations at birth are more likely to be affected by factors, such as the timing of blood collection and temperature (38), further studies are necessary to confirm these results.

The inverse associations between THs and female AGI might be explained by the effects of THs on sex steroid hormone synthesis (1). Female AGD development during the fetal masculinization window was also suggested to be affected by androgen concentrations (39). In *in vitro* studies, THs along with other gonadotropic hormones could inhibit the excessive production of androgens in theca cells isolated from medium-sized follicles (40). Animal studies have also reported that THs could decrease the expression of steroid 5α-reductase type 2, one androgen-related gene, playing anti-androgen effects in the ovary tissue of female S. tropicalis frogs (41). Another mechanism that affecting female AGD through estrogenic pathways has also been proposed (39). THs could also up-regulate the expression of the estrogen receptor alpha and increase the SHBG concentrations in females, resulting in increased serum estrogen concentrations (42, 43). Male reproductive development is intricately dependent on fetal androgen action. Increased fetal androgen concentrations are the main cause for longer AGD in males (44). It is reported that increased TSH concentrations are associated with decreased sex-hormone binding globulin (SHBG) concentrations in males (42), which in turn, will increase the bioavailability of testosterone (43).

Our study has several strengths. First, this longitudinal study used repeated measurements of AGD at multiple ages of early life to examine the association between neonatal thyroid function and AGD, which enabled us to assess the average effect of neonatal thyroid function on AGD development from birth to 48 months of age. Second, the present study was based on a well-designed birth cohort study, which enabled us to obtain information on a wide range of covariates. In addition, the findings were strengthened when similar associations were observed through various analytic strategies.

Several limitations should also be acknowledged in the present study. First, the AGD metrics at each follow-up visit were measured only once, which may have resulted in misclassification. However, it has been reported that the reliability coefficient increased only slightly when the average of two or three repeated measurements for AGD was used (14). In addition, the examiners were blinded to data on neonatal thyroid function, and the misclassification would be non-differential and would bias the associations towards null. Second, the sample size was relatively small, and a total of 80 associations between neonatal thyroid function and AGI were tested using multiple linear regression models in the present study, which may have increased the type I error. However, consistent patterns of inverse associations between neonatal THs and female AGI in GEE models were also observed, which may be less prone to the issue of multiple comparisons when the estimates were calculated for the overall effects from birth to 48 months of age. Third, circulating THs and TSH of both maternal and fetal origin are present in the fetus from the second trimester onward (45). We did not measure maternal TH and TSH concentrations; thus, the contribution of maternal TH and TSH status to our findings cannot be evaluated. Finally, because we measured THs at birth rather than during the fetal masculinization window, reverse causality could probably occur considering that sex steroids can also affect the TH axis. However, the effects of THs on sex steroids synthesis and action are supported by considerable data while the evidence for the opposite direction of crosstalk is much more restricted (1).

In conclusion, inverse associations between neonatal THs and female AGI and a positive association between neonatal TSH and male AGI were indicated. These findings provide additional evidence for the effects of neonatal thyroid function on reproductive development during early childhood.

## Data Availability Statement

The raw data supporting the conclusions of this article will be made available by the authors, without undue reservation.

## Ethics Statement

The studies involving human participants were reviewed and approved by the ethical committee of Shanghai Institute of Planned Parenthood Research. Written informed consent to participate in this study was provided by the participants’ legal guardian/next of kin.

## Author Contributions

ML: data collection, formal analysis, investigation, writing-original draft, and writing-review and editing. HL: design of the data collection instruments, data collection, and writing-review and editing. GHF laboratory analyses, and writing-review and editing. ZW: design of the data collection instruments, data collection, funding acquisition, and writing-review and editing. XS: writing-review and editing. AC: writing-review and editing. MM: study conception and design, project supervision, funding acquisition, and writing-review and editing. WY: study conception and design, project supervision, and writing-review and editing. All authors accept accountability for the overall work. All authors contributed to the article and approved the submitted version.

## Funding

This work was supported by grants from the National key research and development program (2016YFC1000505); National Natural Science Foundation of China (22076123, 81903346); the Science and Technology Commission of Shanghai Municipality (20ZR1448000); Shanghai Sailing Program (18YF1420500).

## Conflict of Interest

The authors declare that the research was conducted in the absence of any commercial or financial relationships that could be construed as a potential conflict of interest.

## Publisher’s Note

All claims expressed in this article are solely those of the authors and do not necessarily represent those of their affiliated organizations, or those of the publisher, the editors and the reviewers. Any product that may be evaluated in this article, or claim that may be made by its manufacturer, is not guaranteed or endorsed by the publisher.
