# Supplementary material for: Association Between Neonatal Thyroid Function and Anogenital Distance from Birth to 48 Months of Age
Source: Front Endocrinol (Lausanne). 2021 Sep 8;12:736505. doi: 10.3389/fendo.2021.736505 (PMC8456038; doi:10.3389/fendo.2021.736505)
Supplement: Supplementary file 1 [file DataSheet_1.docx]

**Supplemental Material**

**Table of Contents**

**Table S1.** *P*-values of interaction item between THs and TSH and child age at follow-up (categorized as birth, 0.5, 1, 2, and 4 years) (exposure*child age at follow-up visit) in the GEE models.

**Table S2.** The baseline characteristics between the included mother-child pairs and those excluded.

**Table S3.** Determinants of neonatal THs and TSH concentrations.

**Table S4.** Associations of neonatal THs and TSH concentrations with AGD in children from birth to 48 months of age in the GEE models.

**Table S5.** Associations of neonatal THs and TSH concentrations with AGI in children with negative TPOAb from birth to 48 months of age in the GEE models.

**Table S6.** Associations of neonatal THs and TSH concentrations with AGI in children when we excluded children with gestational ages <37 weeks in the GEE models.

**Table S7.** Associations of neonatal THs and TSH concentrations with AGI after excluding children whose mothers reported prior or current diagnoses of thyroid-related disease / related medication use in the GEE models.

**Table S8.** Associations of neonatal THs and TSH concentrations with AGI in children whose mothers were normal weight in the GEE models.

**Table S9.** Associations of neonatal THs and TSH concentrations with AGI in children whose mothers were delivered vaginally in the GEE models.

**Figure of contents**

**Figure S1.** The distributions of AGD at birth, and at 6, 12, and 48 months of age.

**Table S1**. *P*-values of interaction item between THs and TSH and child age at follow-up (categorized as birth, 0.5, 1, 2, and 4 years (exposure*child age at follow-up visit) in the GEE models.

|  | Girls | |  | Boys | |
| --- | --- | --- | --- | --- | --- |
|  | AGI_AC_ | AGI_AF_ |  | AGI_AP_ | AGI_AS_ |
| TT_3_ | 0.96 | 0.66 |  | 0.40 | 0.80 |
| TT_4_ | 0.11 | 0.85 |  | **0.04** | 0.19 |
| FT_3_ | 0.99 | 0.80 |  | 0.29 | 0.98 |
| FT_4_ | 0.13 | 0.71 |  | 0.38 | 0.58 |
| TSH (log_10_-transformed) | 0.85 | 0.52 |  | 0.19 | **0.04** |

**Table S2.** The baseline characteristics between the included mother-child pairs and those excluded.

| Characteristics | Included (n=344) | Excluded (n=267) |
| --- | --- | --- |
|  | Mean (SD) / N (%) | Mean (SD)/ N (%) |
| Maternal age (years) | 28.09(3.38) | 28.30(3.27) |
| Gestational age (weeks) | 39.67(1.18) | 39.62(1.29) |
| Pre-pregnancy body mass index (kg/m²) |  |  |
| <18.5 | 68 (20.12) | 62 (23.66) |
| 18.5-24 | 246 (72.78) | 181 (69.08) |
| ≥24 | 24 (7.10) | 19 (7.25) |
| Educational level |  |  |
| High school or below | 75 (21.87) | 66 (24.72) |
| College or above | 268 (78.13) | 201(75.28) |
| Parity^*^ |  |  |
| Nulliparous | 298 (86.88) | 230 (86.47) |
| Multiparous | 45 (13.12) | 36 (13.53) |
| Family income per capita (RMB) |  |  |
| <4000 | 70 (20.47) | 60 (22.81) |
| 4000-8000 | 151 (44.15) | 94 (35.74) |
| ≥8000 | 121 (35.38) | 109 (41.44) |
| Maternal pre-pregnancy passive smoking |  |  |
| Yes | 143 (41.69) | 101 (37.83) |
| No | 200 (58.31) | 166 (62.17) |
| Paternal alcohol consumption before conception |  |  |
| Yes | 112 (32.56) | 81 (30.45) |
| No | 232 (67.44) | 185 (69.55) |
| 5-min Apgar score |  |  |
| 8 | 1 (0.29) | 1 (0.38) |
| 9 | 341 (99.13) | 259 (99.23) |
| 10 | 2 (0.53) | 1 (0.38) |
| Sex |  |  |
| Boys | 194 (56.40) | 132 (49.44) |
| Girls | 150 (43.60) | 135 (50.56) |

Missing data：for the included mother-child pairs：maternal education (n = 1), maternal pre-pregnancy BMI (n = 6), maternal passive smoking (n = 1), parity (n = 1), and family income per capita (n = 2).

For the excluded mother-child pairs: maternal pre-pregnancy BMI (n=5)，parity (n = 1), family income per capita (n = 3), Paternal alcohol consumption before conception (n=1), and 5-min Apgar score (n=6).

^*^ Significance level of the chi-square test

**Table S3.** Determinants of neonatal THs and TSH concentrations.

| Characteristics | N | TSH |  | TT_3_ |  | FT_3_ |  | TT_4_ |  | FT_4_ |
| --- | --- | --- | --- | --- | --- | --- | --- | --- | --- | --- |
|  |  | GM±GSD |  | Mean ± SD |  | Mean ± SD |  | Mean ± SD |  | Mean ± SD |
| Maternal age (year) | | | | | | | | | | |
| <25 | 60 | 2.42 (1.30)^*^ |  | 0.87 (0.15) |  | 1.83 (0.38) |  | 97.23 (20.54) |  | 14.33 (1.57) |
| 25-30 | 195 | 2.23 (1.29) |  | 0.86 (0.15) |  | 1.82 (0.36) |  | 93.14 (28.18) |  | 14.19 (2.07) |
| ≥30 | 89 | 2.18 (1.29) |  | 0.86 (0.18) |  | 1.79 (0.38) |  | 94.17 (26.89) |  | 14.21 (1.90) |
| Maternal pre-pregnancy BMI (kg/m^2)^ | | | | | | | | | | |
| <18.5 | 68 | 2.20 (1.31) |  | 0.83 (0.12) |  | 1.66 (0.28)^*^ | | 83.96 (25.96)^*^ | | 13.46 (1.80)^*^ |
| 18.5~23.9 | 246 | 2.28 (1.29) |  | 0.87 (0.17) |  | 1.86 (0.39) |  | 97.33 (26.42) |  | 14.41 (1.99) |
| ≥24 | 24 | 2.19 (1.24) |  | 0.86 (0.11) |  | 1.81 (0.28) |  | 94.83 (23.87) |  | 14.63 (1.35) |
| Maternal education | | | | | | | | | | |
| High school or below | 75 | 2.36 (1.28) |  | 0.87 (0.14) |  | 1.84 (0.35) |  | 97.63 (22.38) |  | 14.40 (1.64) |
| College or above | 268 | 2.22 (1.30) |  | 0.86 (0.16) |  | 1.81 (0.38) |  | 93.23 (27.67) |  | 14.17 (2.02) |
| Parity | | | | | | | | | | |
| 0 | 298 | 2.25 (1.30) |  | 0.86 (0.15) |  | 1.82 (0.36) |  | 93.65 (26.46) |  | 14.18 (1.96) |
| ≥1 | 45 | 2.24 (1.27) |  | 0.84 (0.20) |  | 1.79 (0.41) |  | 97.46 (28.05) |  | 14.46 (1.84) |
| Maternal depression during pregnancy | | | | | | | | | | |
| No | 276 | 2.26 (1.29) |  | 0.86 (0.16) |  | 1.81 (0.38) |  | 94.18 (27.11) |  | 14.21 (1.96) |
| Yes | 68 | 2.21 (1.34) |  | 0.87 (0.15) |  | 1.85 (0.34) |  | 93.86 (24.77) |  | 14.25 (1.88) |
| Per capita household income (RMB) | | | | | | | | | | |
| <4000 | 70 | 2.28 (1.28) |  | 0.88 (0.19) | | 1.88 (0.46) | | 92.14 (22.16) |  | 14.13 (1.79) |
| 4000-8000 | 151 | 2.29 (1.31) |  | 0.88 (0.14) |  | 1.83 (0.35) |  | 94.75 (28.10) |  | 14.14 (2.00) |
| ≥8000 | 121 | 2.18 (1.29) |  | 0.83 (0.15) |  | 1.76 (0.33) |  | 94.22 (27.39) |  | 14.36 (1.98) |
| Passive smoker before conception | | | | | | | | | | |
| No | 200 | 2.23 (1.30) |  | 0.86 (0.16) |  | 1.78 (0.38)^*^ | | 92.32 (26.09) |  | 14.04 (1.86)^*^ |
| Yes | 143 | 2.29 (1.29) |  | 0.87 (0.15) |  | 1.86 (0.36) |  | 96.63 (27.36) |  | 14.48 (2.04) |
| Mode of delivery | | | | | | | | | | |
| Vaginal delivery | 170 | 2.44 (1.31)^*^ |  | 0.84 (0.15)^*^ | | 1.75 (0.37)^*^ | | 93.78 (26.11) |  | 13.96 (1.88)^*^ |
| Cesarean section | 172 | 2.07 (1.24) |  | 0.88 (0.16) |  | 1.88 (0.36) |  | 94.43 (26.09) |  | 14.47 (1.86) |
| Paternal age (year) | | | | | | | | | | |
| <25 | 27 | 2.68 (1.31)^*^ |  | 0.84 (0.13) |  | 1.82 (0.29) |  | 94.16 (21.54) |  | 14.12 (1.66) |
| 25-30 | 139 | 2.20 (1.29) |  | 0.87 (0.16) |  | 1.82 (0.38) |  | 93.12 (25.31) |  | 14.18 (1.98) |
| ≥30 | 177 | 2.23 (1.28) |  | 0.85 (0.16) |  | 1.80 (0.36) |  | 94.71 (28.36) |  | 14.24 (1.94) |
| Paternal BMI (kg/m^2^) | | | | | | | | | | |
| <18.5 | 14 | 2.11 (1.26) |  | 0.85 (0.07) |  | 1.75 (0.25) |  | 94.39 (31.40) |  | 13.98 (2.02) |
| 18.5~23.9 | 189 | 2.25 (1.30) |  | 0.86 (0.15) |  | 1.81 (0.37) |  | 92.67 (27.12) |  | 14.11 (1.97) |
| ≥24 | 125 | 2.27 (1.29) |  | 0.87 (0.17) |  | 1.84 (0.37) |  | 97.04 (25.79) |  | 14.47 (1.91) |
| Paternal education | | | | | | | | | | |
| High school or below | 70 | 2.28 (1.30) |  | 0.87 (0.15) |  | 1.85 (0.37) |  | 91.52 (23.29) |  | 14.02 (1.86) |
| College or above | 274 | 2.24 (1.29) |  | 0.86 (0.16) |  | 1.81 (0.37) |  | 94.78 (27.42) |  | 14.27 (1.97) |
| Paternal smoking within 3 months before conception | | | | | | | | | | |
| No | 240 | 2.26 (1.30) |  | 0.86 (0.16) |  | 1.81 (0.38) |  | 93.54 (26.95) |  | 14.21 (1.97) |
| Yes | 104 | 2.22 (1.29) |  | 0.87 (0.16) |  | 1.84 (0.35) |  | 95.45 (25.97) |  | 14.25 (1.90) |
| Paternal alcohol within months before conception | | | | | | | | | | |
| No | 232 | 2.20 (1.29)^*^ |  | 0.86 (0.15) |  | 1.79 (0.37)^*^ | | 92.24 (27.04) | | 14.10 (1.93) |
| Yes | 112 | 2.35 (1.30) |  | 0.87 (0.16) |  | 1.88 (0.37) |  | 98.01 (25.44) |  | 14.47 (1.96) |
| Infant sex | | | | | | | | | | |
| Boy | 194 | 2.26 (1.28) |  | 0.85 (0.16) |  | 1.78 (0.37) | | 92.91 (26.89) |  | 14.19 (1.92) |
| Girl | 150 | 2.23 (1.32) |  | 0.87 (0.16) |  | 1.86 (0.37) |  | 95.68 (26.3) |  | 14.26 (1.98) |
| Gestational weeks |  |  |  |  |  |  |  |  |  |  |
| < 37 | 5 | 2.49 (1.26) |  | 0.86 (0.16) |  | 1.87 (0.48) |  | 109.4 (30.98) |  | 15.41 (3.27) |
| ≥ 37 | 339 | 2.25 (1.30) |  | 0.87 (0.21) |  | 1.81 (0.37) |  | 93.90 (26.55) |  | 14.20 (1.92) |

Missing data： maternal education (n = 1), maternal pre-pregnancy BMI (n = 6), maternal passive smoking (n = 1), parity (n = 1), race (n=2), and family income per capita (n = 2).

^*^Statistically significant difference (*p* < 0.05)

**Table S4.** Associations of neonatal THs and TSH concentrations with AGD in children from birth to 48 months of age in the GEE models.

| THs and TSH | Girls | |  | Boys | |
| --- | --- | --- | --- | --- | --- |
|  | AGD_AC_ | AGD_AF_ |  | AGD_AP_ | AGD_AS_ |
| TT_3_ | | | | | |
| Lowest tertile | Ref | Ref |  | Ref | Ref |
| Middle tertile | -1.03 (-2.73, 0.66) | -1.17 (-2.26, -0.08) |  | -0.15 (-1.88, 1.57) | 0.77 (-1.16, 2.70) |
| Highest tertile | -0.59 (-2.56, 1.38) | -0.78 (-2.02, 0.45) |  | 0.30 (-1.41, 2.00) | 0.67 (-1.22, 2.55) |
| TT_4_ | | | | | |
| Lowest tertile | Ref | Ref |  | Ref | Ref |
| Middle tertile | **-2.41 (-4.14, -0.67)^*^** | **-1.52 (-2.76, -0.28)^*^** | -0.60 (-2.36, 1.16) | -0.60 (-2.36, 1.16) | -0.36 (-2.29, 1.57 |
| Highest tertile | -1.45 (-3.31, 0.42) | -0.93 (-2.21, 0.34) | -0.69 (-2.38, 0.99) | -0.69 (-2.38, 0.99) | -0.15 (-1.98, 1.68) |
| FT_3_ | | | | | |
| Lowest tertile | Ref | Ref |  | Ref | Ref |
| Middle tertile | **-2.09 (-4.05, -0.12)^*^** | **-1.28 (-2.52, -0.05)^*^** |  | -0.10 (-1.95, 1.75) | 0.56 (-1.46, 2.59) |
| Highest tertile | **-2.35 (-4.31, -0.39)^*^** | **-2.04 (-3.27, -0.80)^*^** |  | -0.91 (-2.64, 0.82) | -0.71 (-2.65, 1.23) |
| FT_4_ | | | | | |
| Lowest tertile | Ref | Ref |  | Ref | Ref |
| Middle tertile | **-2.76 (-4.68, -0.84)^*^** | **-1.70 (-3.07, -0.32)^*^** |  | -1.44 (-3.17, 0.28) | -1.13 (-3.00, 0.73) |
| Highest tertile | -1.69 (-3.56, 0.19)^#^ | -1.04 (-2.31, 0.23) |  | -1.09 (-2.87, 0.70) | -0.97 (-3.00, 1.07) |
| TSH (log_10_-transformed) | | | | | |
| Lowest tertile | Ref | Ref |  | Ref | Ref |
| Middle tertile | -1.07 (-2.86, 0.72) | -1.18 (-2.40, 0.04)^#^ |  | 0.21 (-1.59, 2.01) | 0.52 (-1.39, 2.44) |
| Highest tertile | -1.22 (-3.10, 0.66) | -0.44 (-1.70, 0.82) |  | 0.94 (-0.77, 2.65) | 1.45 (-0.42, 3.31) |

Adjusted for maternal age, maternal education, maternal pre-pregnancy BMI, gestational weeks, maternal passive smoking, paternal alcohol consumption, child’s age, and weight-for-length z scores.

^*^Statistically significant difference (*p* < 0.05)

^#^Marginally significant difference (*p* < 0.10).

**Table S5.** Associations of neonatal THs and TSH concentrations with AGI in children with negative TPOAb from birth to 48 months of age in the GEE models.

| THs and TSH | Girls | |  | Boys | |
| --- | --- | --- | --- | --- | --- |
|  | AGI_AC_ | AGI_AF_ |  | AGI_AP_ | AGI_AS_ |
| TT_3_ | | | | | |
| Lowest tertile | Ref | Ref |  | Ref | Ref |
| Middle tertile | 0.13 (-0.11, 0.37) | -0.07 (-0.22, 0.09) |  | 0.04 (-0.22, 0.30) | 0.12 (-0.11, 0.34) |
| Highest tertile | 0.12 (-0.15, 0.39) | -0.03 (-0.19, 0.13) |  | 0.11 (-0.16, 0.38) | 0.14 (-0.09, 0.38) |
| TT_4_ | | | | | |
| Lowest tertile | Ref | Ref |  | Ref | Ref |
| Middle tertile | **-0.27 (-0.51, -0.03)^*^** | -0.12 (-0.29, 0.04) |  | -0.14 (-0.41, 0.13) | -0.05 (-0.29, 0.19) |
| Highest tertile | -0.20 (-0.44, 0.04) | -0.07 (-0.23, 0.09) |  | -0.06 (-0.33, 0.22) | -0.01 (-0.23, 0.21) |
| FT_3_ | | | | | |
| Lowest tertile | Ref | Ref |  | Ref | Ref |
| Middle tertile | -0.03 (-0.28, 0.22) | -0.08 (-0.24, 0.08) |  | 0.10 (-0.17, 0.37) | 0.10 (-0.13, 0.33) |
| Highest tertile | -0.08 (-0.33, 0.17) | **-0.21 (-0.36, -0.07)^*^** |  | -0.07 (-0.34, 0.19) | -0.08 (-0.32, 0.15) |
| FT_4_ | | | | | |
| Lowest tertile | Ref | Ref |  | Ref | Ref |
| Middle tertile | **-0.38 (-0.62, -0.13)^*^** | **-0.16 (-0.34, 0.02)^#^** |  | -0.14 (-0.41, 0.12) | -0.09 (-0.32, 0.15) |
| Highest tertile | **-0.28 (-0.56, 0.01)^#^** | -0.11 (-0.29, 0.07) |  | -0.03 (-0.31, 0.25) | 0.02 (-0.24, 0.27) |
| TSH (log 10-transformed) | | | | | |
| Lowest tertile | Ref | Ref |  | Ref | Ref |
| Middle tertile | -0.04 (-0.28, 0.19) | -0.03 (-0.18, 0.13) |  | 0.03 (-0.25, 0.32) | 0.14 (-0.10, 0.38) |
| Highest tertile | 0.04 (-0.24, 0.31) | 0.11 (-0.06, 0.27) |  | 0.09 (-0.19, 0.36) | **0.20 (-0.04, 0.43)^#^** |

Adjusted for maternal age, maternal education, maternal pre-pregnancy BMI, gestational weeks, maternal passive smoking, child’s age, and paternal alcohol consumption.

^*^Statistically significant difference (*p* < 0.05)

^#^Marginally significant difference (*p* < 0.10).

**Table S6.** Associations of neonatal THs and TSH concentrations with AGI in children when we excluded children with gestational ages <37 weeks in the GEE models.

| THs and TSH | Girls | |  | Boys | |
| --- | --- | --- | --- | --- | --- |
|  | AGI_AC_ | AGI_AF_ |  | AGI_AP_ | AGI_AS_ |
| TT_3_ | | | | | |
| Lowest tertile | Ref | Ref |  | Ref | Ref |
| Middle tertile | 0.06 (-0.17, 0.29) | -0.07 (-0.22, 0.08) |  | 0.09 (-0.18, 0.36) | 0.16 (-0.07, 0.39) |
| Highest tertile | 0.03 (-0.24, 0.29) | -0.04 (-0.19, 0.12) |  | 0.13 (-0.16, 0.41) | 0.12 (-0.12, 0.36) |
| TT_4_ | | | | | |
| Lowest tertile | Ref | Ref |  | Ref | Ref |
| Middle tertile | **-0.26 (-0.49, -0.03)^*^** | **-0.14 (-0.30, 0.02)^#^** |  | -0.2 (-0.48, 0.08) | -0.09 (-0.33, 0.16) |
| Highest tertile | **-0.26 (-0.50, -0.02)^*^** | -0.09 (-0.25, 0.06) |  | -0.02 (-0.30, 0.27) | 0.01 (-0.20, 0.23) |
| FT_3_ | | | | | |
| Lowest tertile | Ref | Ref |  | Ref | Ref |
| Middle tertile | -0.10 (-0.35, 0.14) | -0.08 (-0.23, 0.07) |  | 0.12 (-0.16, 0.39) | 0.10 (-0.14, 0.34) |
| Highest tertile | -0.18 (-0.43, 0.07) | **-0.22 (-0.36, -0.08)^*^** |  | 0 (-0.28, 0.28) | -0.05 (-0.29, 0.19) |
| FT_4_ | | | | | |
| Lowest tertile | Ref | Ref |  | Ref | Ref |
| Middle tertile | **-0.39 (-0.61, -0.17)^*^** | **-0.15 (-0.32, 0.01)^#^** |  | -0.14 (-0.41, 0.14) | -0.07 (-0.31, 0.16) |
| Highest tertile | **-0.31 (-0.57, -0.06)^*^** | -0.11 (-0.28, 0.06) |  | -0.02 (-0.32, 0.29) | -0.01 (-0.26, 0.24) |
| TSH (log_10_-transformed) | | | | | |
| Lowest tertile | Ref | Ref |  | Ref | Ref |
| Middle tertile | -0.07 (-0.30, 0.17) | -0.05 (-0.19, 0.10) |  | 0.03 (-0.27, 0.32) | 0.14 (-0.10, 0.38) |
| Highest tertile | 0.05 (-0.22, 0.31) | 0.11 (-0.05, 0.26) |  | 0.14 (-0.17, 0.45) | 0.22 (-0.02, 0.46) |

Adjusted for maternal age, maternal education, maternal pre-pregnancy BMI, gestational weeks, maternal passive smoking, child’s age, and paternal alcohol consumption.

^*^Statistically significant difference (*p* < 0.05)

^#^Marginally significant difference (*p* < 0.10).

**Table S7.** Associations of neonatal THs and TSH concentrations with AGI after excluding children whose mothers reported prior or current diagnoses of thyroid-related disease / related medication use in the GEE models.

| THs and TSH | Girls | |  | Boys | |
| --- | --- | --- | --- | --- | --- |
|  | AGI_AC_ | AGI_AF_ |  | AGI_AP_ | AGI_AS_ |
| TT_3_ | | | | | |
| Lowest tertile | Ref | Ref |  | Ref | Ref |
| Middle tertile | 0.04 (-0.21, 0.28) | -0.10 (-0.24, 0.04) |  | 0.12 (-0.14, 0.39) | 0.14 (-0.09, 0.36) |
| Highest tertile | 0.04 (-0.24, 0.32) | -0.03 (-0.19, 0.12) |  | 0.21 (-0.08, 0.49) | 0.11 (-0.12, 0.34) |
| TT4 | | | | | |
| Lowest tertile | Ref | Ref |  | Ref | Ref |
| Middle tertile | **-0.35 (-0.59, -0.10)^*^** | **-0.16 (-0.31, -0.01)^*^** |  | -0.22 (-0.5, 0.06) | -0.06 (-0.3, 0.18) |
| Highest tertile | -0.12 (-0.36, 0.12) | -0.09 (-0.24, 0.06) |  | 0.01 (-0.28, 0.30) | 0.04 (-0.17, 0.25) |
| FT3 | | | | | |
| Lowest tertile | Ref | Ref |  | Ref | Ref |
| Middle tertile | -0.09 (-0.35, 0.17) | -0.08 (-0.23, 0.07) |  | 0.06 (-0.22, 0.33) | 0.09 (-0.14, 0.33) |
| Highest tertile | -0.15 (-0.41, 0.12) | **-0.20 (-0.34, -0.06)^*^** |  | -0.02 (-0.3, 0.26) | -0.06 (-0.29, 0.17) |
| FT4 | | | | | |
| Lowest tertile | Ref | Ref |  | Ref | Ref |
| Middle tertile | **-0.28 (-0.52, -0.05)^*^** | **-0.17 (-0.33, -0.01)^*^** |  | -0.13 (-0.38, 0.13) | -0.07 (-0.29, 0.16) |
| Highest tertile | -0.18 (-0.45, 0.09) | -0.12 (-0.29, 0.04) |  | -0.03 (-0.32, 0.27) | -0.01 (-0.25, 0.22) |
| TSH (log10-transformed) | | | | | |
| Lowest tertile | Ref | Ref |  | Ref | Ref |
| Middle tertile | 0.07 (-0.21, 0.35) | -0.03 (-0.17, 0.11) |  | 0.02 (-0.28, 0.31) | 0.15 (-0.08, 0.38) |
| Highest tertile | 0.08 (-0.19, 0.35) | 0.11 (-0.04, 0.27) |  | 0.06 (-0.23, 0.35) | **0.22 (-0.01, 0.44)^#^** |

Adjusted for maternal age, maternal education, maternal pre-pregnancy BMI, gestational weeks, maternal passive smoking, child’s age, and paternal alcohol consumption.

^*^Statistically significant difference (*p* < 0.05)

^#^Marginally significant difference (*p* < 0.10).

**Table S8.** Associations of neonatal THs and TSH concentrations with AGI in children whose mothers were normal weight in the GEE models.

| THs and TSH | Girls | |  | Boys | |
| --- | --- | --- | --- | --- | --- |
|  | AGI_AC_ | AGI_AF_ |  | AGI_AP_ | AGI_AS_ |
| TT_3_ | | | | | |
| Lowest tertile | Ref | Ref |  | Ref | Ref |
| Middle tertile | 0.11 (-0.17, 0.40) | -0.01 (-0.19, 0.17) |  | -0.01 (-0.34, 0.33) | 0.07 (-0.22, 0.35) |
| Highest tertile | 0.08 (-0.24, 0.41) | -0.02 (-0.20, 0.16) |  | 0.10 (-0.23, 0.43) | 0.04 (-0.24, 0.33) |
| TT_4_ | | | | | |
| Lowest tertile | Ref | Ref |  | Ref | Ref |
| Middle tertile | **-0.37 (-0.67, -0.06)^*^** | -0.14 (-0.36, 0.07) |  | -0.09 (-0.43, 0.25) | -0.01 (-0.31, 0.29) |
| Highest tertile | -0.22 (-0.52, 0.08) | -0.08 (-0.29, 0.12) |  | 0.13 (-0.23, 0.49) | 0.13 (-0.14, 0.39) |
| FT_3_ | | | | | |
| Lowest tertile | Ref | Ref |  | Ref | Ref |
| Middle tertile | -0.06 (-0.37, 0.24) | -0.07 (-0.26, 0.12) |  | 0.03 (-0.31, 0.38) | 0 (-0.30, 0.30) |
| Highest tertile | -0.18 (-0.47, 0.11) | **-0.28 (-0.44, -0.11)^*^** |  | 0.08 (-0.23, 0.39) | 0 (-0.27, 0.27) |
| FT_4_ | | | | | |
| Lowest tertile | Ref | Ref |  | Ref | Ref |
| Middle tertile | **-0.54 (-0.84, -0.24)^*^** | **-0.21 (-0.43, 0.02)^#^** |  | -0.02 (-0.33, 0.28) | -0.02 (-0.29, 0.25) |
| Highest tertile | **-0.40 (-0.71, -0.09)^*^** | -0.14 (-0.35, 0.07) |  | 0.1 (-0.26, 0.46) | 0.04 (-0.25, 0.34) |
| TSH (log_10-_transformed) | | | | | |
| Lowest tertile | Ref | Ref |  | Ref | Ref |
| Middle tertile | -0.08 (-0.36, 0.21) | -0.03 (-0.22, 0.15) |  | 0.12 (-0.21, 0.46) | 0.18 (-0.10, 0.45) |
| Highest tertile | 0.08 (-0.26, 0.41) | 0.10 (-0.09, 0.29) |  | 0.27 (-0.07, 0.6) | **0.33 (0.07, 0.60)^*^** |

Adjusted for maternal age, maternal education, maternal pre-pregnancy BMI, gestational weeks, maternal passive smoking, child’s age, and paternal alcohol consumption.

^*^Statistically significant difference (*p* < 0.05)

^#^Marginally significant difference (*p* < 0.10).

**Table S9.** Associations of neonatal THs and TSH concentrations with AGI in children whose mothers were delivered vaginally in the GEE models.

| THs and TSH | Girls | |  | Boys | |
| --- | --- | --- | --- | --- | --- |
|  | AGI_AC_ | AGI_AF_ |  | AGI_AP_ | AGI_AS_ |
| TT_3_ | | | | | |
| Lowest tertile | Ref | Ref |  | Ref | Ref |
| Middle tertile | 0.10 (-0.22, 0.42) | -0.01 (-0.23, 0.21) |  | 0.15 (-0.18, 0.48) | 0.18 (-0.14, 0.49) |
| Highest tertile | 0.32 (-0.08, 0.72) | 0.11 (-0.15, 0.38) |  | 0.27 (-0.16, 0.69) | 0.07 (-0.28, 0.41) |
| TT_4_ | | | | | |
| Lowest tertile | Ref | Ref |  | Ref | Ref |
| Middle tertile | **-0.31 (-0.66, 0.04)^#^** | -0.18 (-0.46, 0.09) |  | -0.16 (-0.49, 0.16) | -0.03 (-0.36, 0.31) |
| Highest tertile | -0.30 (-0.70, 0.10) | -0.2 (-0.47, 0.07) |  | -0.28 (-0.67, 0.11) | -0.18 (-0.49, 0.13) |
| FT_3_ | | | | | |
| Lowest tertile | Ref | Ref |  | Ref | Ref |
| Middle tertile | -0.06 (-0.42, 0.30) | -0.21 (-0.43, 0.01) |  | 0.02 (-0.30, 0.34) | 0.15 (-0.14, 0.44) |
| Highest tertile | -0.02 (-0.48, 0.43) | **-0.23 (-0.44, -0.03)^*^** |  | 0.18 (-0.25, 0.61) | -0.03 (-0.4, 0.34) |
| FT_4_ | | | | | |
| Lowest tertile | Ref | Ref |  | Ref | Ref |
| Middle tertile | **-0.48 (-0.83, -0.13)^*^** | -0.09 (-0.34, 0.16) |  | -0.05 (-0.37, 0.27) | -0.16 (-0.45, 0.14) |
| Highest tertile | -0.19 (-0.61, 0.23) | -0.11 (-0.36, 0.16) |  | -0.02 (-0.41, 0.37) | -0.07 (-0.42, 0.28) |
| TSH (log_10_-transformed) | | | | | |
| Lowest tertile | Ref | Ref |  | Ref | Ref |
| Middle tertile | -0.17 (-0.58, 0.25) | -0.02 (-0.29, 0.25) |  | 0 (-0.38, 0.39) | 0.27 (-0.06, 0.61) |
| Highest tertile | -0.20 (-0.57, 0.18) | 0 (-0.25, 0.25) |  | 0.18 (-0.19, 0.56) | 0.23 (-0.07, 0.53) |

Adjusted for maternal age, maternal education, maternal pre-pregnancy BMI, gestational weeks, maternal passive smoking, child’s age, and paternal alcohol consumption.

^*^Statistically significant difference (*p* < 0.05)

^#^Marginally significant difference (*p* < 0.10).

**Figure S1.** The distributions of AGD at birth, and at 6, 12, and 48 months of age.
